# A Comprehensive Analysis of Microbial Community and Nitrogen Removal Rate Predictions in Three Anammox Systems

**DOI:** 10.3390/microorganisms13122795

**Published:** 2025-12-08

**Authors:** Xuan Zhang, Tao Ya, Lu Han, Weize Li

**Affiliations:** State Key Laboratory of Environmental Criteria and Risk Assessment, Chinese Research Academy of Environmental Sciences, Beijing 100012, China; zhangxuan244@mails.ucas.ac.cn (X.Z.); yatao@craes.org.cn (T.Y.); 18204786595@163.com (W.L.)

**Keywords:** anammox, microbial community, system type, machine learning, 16S rRNA gene

## Abstract

Anammox is a promising approach for biological nitrogen removal, but the differences in microbial community structure across different systems and their response mechanisms to environmental factors remain unclear. In this study, 206 microbial samples and 2126 environmental factor data points from three different anammox systems, including the upflow anaerobic sludge blanket (UASB), integrated fixed-film activated sludge-partial nitritation/anammox (IFAS-PN/A), and integrated fixed-film activated sludge-simultaneous nitrification, anammox and denitrification (IFAS-SNAD), were analyzed using 16S rRNA sequencing analysis, bioinformatics, and machine learning (ML) techniques. The results revealed significant differences in microbial composition among three systems, evidenced by the enrichment of *Candidatus_Brocadia* in IFAS-PN/A, the high-diversity community in IFAS-SNAD, and the low-diversity communities dominated by *Candidatus_Kuenenia* in the UASB. Co-occurrence network analysis demonstrated more tightly connected and complex interactions in IFAS-SNAD networks. Machine learning predictions further showed that the stacked model (ST-RF) achieved the highest accuracy in predicting the nitrogen removal rate (NRR), with determination coefficients (R^2^) exceeding 0.987 across all testing datasets. Moreover, SHapley Additive exPlanations (SHAP) analysis based on the stacked model revealed that the influence of key environmental factors on NRR varied by system type. These results suggested that NRR of different systems depended on the control of key environmental factors, while the significance of these environmental factors was determined by the type of system. Overall, this study enhanced the ecological and functional understanding of anammox-based processes and provided a data-driven framework for optimizing mainstream nitrogen removal.

## 1. Introduction

Anaerobic ammonia oxidation (anammox) process has emerged as an effective and environmentally sustainable approach for nitrogen removal [1,2,3]. This process is primarily driven by anammox bacteria (AnAOB), which convert ammonium nitrogen (NH_4_^+^-N) directly into nitrogen gas (N_2_) by employing nitrite nitrogen (NO_2_^−^-N) as the electron acceptor, thereby eliminating the need for aeration or external organic carbon [4,5]. However, AnAOB has been observed to exhibit a relatively slow growth rate, with a doubling time of 10–12 days under optimal conditions [6,7]. And it is known to be sensitive to changes in environmental conditions, including dissolved oxygen (DO), organic carbon levels, ammonium concentrations, and temperature. This sensitivity renders it challenging for AnAOB to be retained and enriched within treatment systems [8]. Additionally, AnAOB competes with rapidly growing nitrite-oxidizing bacteria (NOB) for both substrate and space [9]. These challenges are significant barriers to the implementation of anammox technology in wastewater treatment facilities.

To overcome these barriers, various reactor configurations have been designed, including the upflow anaerobic sludge blanket (UASB), integrated fixed-film activated sludge-partial nitritation/anammox (IFAS-PN/A), and integrated fixed-film activated sludge-simultaneous nitrification, anammox, and denitrification (IFAS-SNAD) systems. Each configuration creates distinct ecological conditions that influence microbial enrichment and nitrogen transformation pathways. Among these, the IFAS-PN/A process enhances nitrogen removal by selectively retaining AnAOB in biofilms while suppressing NOB through floc discharge [10,11]. Yang et al. [12] established IFAS-PN/A reactor with a stable nitrogen removal rate (NRR) of 2.0–2.5 kg N/(m^3^·d), with 85% of nitrogen removal efficiency (NRE), attributing this performance to the combined enrichment of ammonium-oxidizing bacteria (AOB) and AnAOB in both flocs and biofilms. Further, Yang et al. [13] demonstrated that IFAS-PN/A system enhanced both the abundance and metabolic activity of functional microorganisms, effectively limiting nitrate accumulation and attaining a maximum NRR of 1.5 kg N/(m^3^·d) with an NRE of 82.3%, in which *Candidatus_Brocadia* was identified as the dominant anammox genus. Building upon the SNAD process, the IFAS-SNAD process enhanced nitrogen removal through the synergistic action of simultaneous nitrification, anammox, and denitrification. Recent studies have clarified the underlying mechanisms of this synergy. For instance, Wang et al. [14] and Du et al. [15] demonstrated that AnAOB were predominantly enriched in the biofilm, working synergistically with heterotrophic denitrifiers and AOB in the suspended sludge. This partitioning enabled high total nitrogen (TN) removal efficiencies (72–92.8%) at optimized operational conditions, showcasing the highly efficient nitrogen removal performance of the IFAS-SNAD system. In contrast to the two IFAS systems, UASB reactor is extensively applied for conducting the anammox process because its sludge readily forms dense granules, facilitating biomass retention and typically resulting in a higher NRR [16,17,18,19]. This system excels at fostering a dominant anammox community, as evidenced by Li et al. [20], where *Candidatus_Kuenenia* proliferated from 0.01% to over 50% abundance, enabling exceptional nitrogen removal efficiencies exceeding 99.6%. Although the performance of anammox sludge formed in these systems has been studied, a systematic comparison of microbial community structures across these systems remains limited.

Predicting nitrogen removal performance in biological processes is difficult because of the interactions between microbial communities and surrounding environmental conditions. Machine learning (ML), recognized as an advanced technique within data mining, has captured considerable interest recently. Previous studies have primarily applied single models to predict nitrogen removal [21,22,23]. For example, a meta-analysis across wastewater treatment plants worldwide used six ML models to predict nitrogen removal, and found that Extreme Gradient Boosting (XGBoost) yielded the highest R^2^ among them, exceeding 0.8 [24]. Another study by Mu et al. [25] showed that while Artificial Neural Network (ANN) and XGBoost demonstrated strong predictive capabilities for NRR and functional microbial abundance, respectively, Support Vector Regression (SVR) performed less effectively, because it struggled to represent intricate nonlinear patterns and was prone to interference from redundant variables. However, single models may be overly sensitive to characteristics and noise in the training data, leading to overfitting issues. Stacked models, which integrate multiple base learners, are regarded as a promising approach by mitigating overfitting and enhancing predictive stability and accuracy [26]. Over the last decade, model stacking has found widespread application across various domains [27,28,29]. Research by Lukas et al. has demonstrated that stacked models exhibit greater stability in small datasets compared to individual ML models [30]. Nonetheless, the application of a stacked model to simulate nitrogen removal process has been relatively uncommon.

Therefore, this study aims to systematically compare the microbial community structures and microbial networks across three anammox systems (IFAS-PN/A, IFAS-SNAD, and UASB) using 16S rRNA gene sequences based on big-data mining, while a stacked model was employed to predict NRR and interpret the key influencing environmental factors through SHapley Additive exPlanations (SHAP). By integrating multiple analytical approaches and machine learning techniques for high throughput sequencing interpretation, this study enhances the precision and efficiency of NRR prediction and offers valuable insights to optimize nitrogen removal processes in wastewater treatment systems.

## 2. Materials and Methods

### 2.1. Collection and Processing of Literature Data

Studies published between 2016 and 2025 were retrieved from Web of Science using keyword combinations of “anammox” with “UASB,” “integrated fixed-film activated sludge (IFAS),” “partial nitritation/anammox (PN/A),” or “simultaneous nitrification, anammox, and denitrification (SNAD).” Only Illumina-based 16S rRNA amplicon datasets with publicly available FASTQ files, complete metadata, and clearly reported primers were included. A screening diagram is provided in Supplementary Figure S1. Comprehensive metadata and accession numbers associated with sequencing datasets collected from National Center for Biotechnology Information (NCBI) Sequence Read Archive are listed in Supplementary Table S1 [31]. A total of 206 samples were collected, including IFAS-PN/A (55 samples), IFAS-SNAD (86 samples), and UASB (65 samples).

Machine learning approaches were additionally utilized in this study to determine which environmental factors exerted significant effects on NRR. In total, 2126 environmental factor data points from the figures of the aforementioned studies were collected in the three systems using Origin software (version 2022) (see Supplementary Materials), including 584 from the IFAS-PN/A system, 420 from the IFAS-SNAD system, and 1122 from the UASB system. Environmental factor data were collected for different reaction systems. At the same time, the corresponding operational variables were sorted out. To minimize the impact of random errors on the extracted data, three extractions were performed at each sampling point, with the final average value used as the experimental analysis data. In this study, the nitrogen loading rate (NLR) and NRR were determined based on the formulas presented in Equations (1) to (4).

(1) $$TN_{inf}\left(mg/L\right)=NH_{4}^{+}-N_{inf}+NO_{2}^{-}{-N}_{inf}+NO_{3}^{-}-N_{inf}$$

(2) $$TN_{eff}\left(mg/L\right)=NH_{4}^{+}-N_{eff}+NO_{2}^{-}-N_{eff}+NO_{3}^{-}-N_{eff}$$

(3) $$NLR\left(\frac{\mathrm{kgN}}{m^{3}\cdot d}\right)=\frac{TN_{\inf}}{1000}\times \frac{24}{\mathrm{HRT}}$$

(4) $$NRR\left(\frac{\mathrm{kgN}}{m^{3}\cdot d}\right)=\frac{TN_{\inf}-TN_{e\mathrm{ff}}}{1000}\times \frac{24}{\mathrm{HRT}}$$

where NH_4_^+^-N_inf_, NO_2_^−^-N_inf_, NO_3_^−^-N_inf_, NH_4_^+^-N_eff_, NO_2_^−^-N_eff_, and NO_3_^−^-N_eff_ are the nitrogen concentrations in mg/L of the influent and effluent, respectively.

### 2.2. Bioinformatics Analysis

All 16S rRNA gene sequences collected from the corresponding literature were grouped by primer type. Each group independently applied Cutadapt v.5.0 [32] and DADA2 plugin [33] to finish removing primers and quality control in QIIME 2 (version 2025.4.0) [34]. Quality control thresholds were enforced during this process. Sequences were filtered using a minimum Phred quality score of 33. Paired-end reads were retained only if the merging success rate exceeded 90%. After processing, representative sequences and feature tables from different groups were merged using the merge-seqs and merge functions of the QIIME 2 plugin. The resulting consolidated dataset was then subjected to taxonomic classification of each amplicon sequence variant (ASV) using the Silva 138 Classifier [35]. Bacterial composition profiles at genus level were subsequently compiled for integrated statistical analyses and visualization. The finalized ASV table formed the basis for later machine learning modeling development.

### 2.3. Diversity Analysis, Network Analysis and Data Visualization

Alpha diversity across the distinct treatment systems was evaluated in R (version 4.5.0) using the vegan v2.6-8, ggplot2 v3.5.1, and agricolae v1.3-7 packages to visualize and compare species richness and Simpson Diversity index. Statistical significance among groups was determined through the Mann–Whitney U test. Community structure differentiation was further examined via Principal Coordinates Analysis (PCoA) and Adonis tests based on Bray–Curtis dissimilarities, both executed with the vegan package.

Co-occurrence networks were generated in R (version 4.5.0) utilizing the igraph v2.1.4, Hmisc v5.2.3, and ggplot2 v3.5.1 packages. To eliminate low-frequency ASVs, only those representing more than 0.01% of the total sequences and appearing in over 20% of samples were retained. Pairwise Spearman’s correlations between ASVs were calculated, with a correlation coefficient > |0.6| and a *p* value < 0.01 (Benjamini and Hochberg adjusted) being considered as a valid relationship [36]. The network-level (average clustering coefficient, modularity, density, and average degree) and node-level (degree, nodes, and edges) topological features of a network were calculated. These networks were visualized using Gephi (version 0.10.1) and were accordingly named IFAS-PN/A, IFAS-SNAD, and UASB.

### 2.4. Machine Learning Model

**Figure 1:**
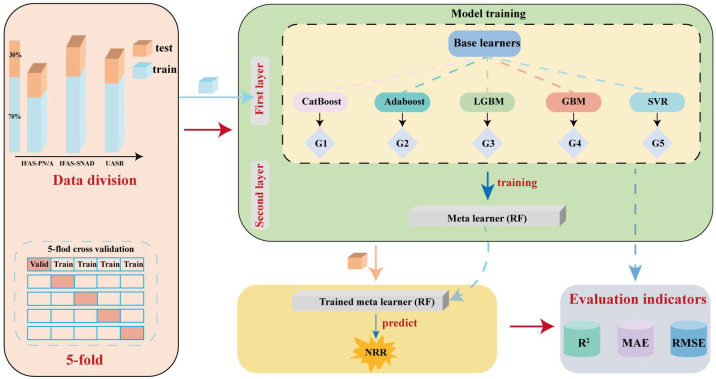
Overall structure of stacked model. Red squares denote the validation set for the current iterative round.

Stacking is a specific type of ensemble machine learning algorithm that can produce a strong learner from poorer ones [37]. To enhance overall performance, this hierarchical approach employs a meta model that synthesizes the outcomes of base models, thereby improving the accuracy and generalization capability of the model. The detailed architecture of the model is shown in Figure 1. The dataset collected was split into training and test sets. Base learners were trained on the training set and their results on the training set served as inputs to a meta learner, which was used to train the final meta learner. After model training, the testing set served as inputs to the meta learner, which generated the final labels. Model performance and hyperparameters were optimized using grid search with five-fold cross-validation, ensuring high accuracy and robustness in practical applications.

In the study, datasets for IFAS-PN/A (*n* = 584), IFAS-SNAD (*n* = 420), and UASB (*n* = 1122) were each divided into training and testing sets at a ratio of 7:3, adhering to the no-peeking principle to prevent data leakage [38,39]. Categorical Boosting (CatBoost), Adaptive Boosting (AdaBoost), Light Gradient Boosting Machine (LGBM), Gradient Boosting Machine (GBM), and SVR were used as base learners, while XGBoost and Random Forest (RF) were served as meta learners to generate two stacked models, namely Stacked-XGBoost (ST-XG) and Stacked-RF (ST-RF). Model performance was evaluated using determination coefficients (R^2^), root mean square error (RMSE), and mean absolute error (MAE) according to Equations (5) to (7), with hyperparameters optimized via five-fold cross-validation and grid search [40,41]. Regularization was applied to all base learners, and outlier problems were implicitly addressed through robust model design and cross-validation.

(5) $$R^{2}=1-\frac{\sum_{i=1}^{N}{\left(p_{i}-\hat{p_{i}}\right)}^{2}}{\sum_{i=1}^{N}{\left(p_{i}-\bar{p}\right)}^{2}}$$

(6) $$RMSE=\sqrt{\frac{1}{N}\sum_{i=1}^{N}{\left(p_{i}-\hat{p_{i}}\right)}^{2}}$$

(7) $$MAE=\frac{1}{N}\sum_{i=1}^{N}\left|p_{i}-\hat{p_{i}}\right|$$

where $\hat{p_{i}}$ is the predicted NRR value, $p_{i}$ is the actual NRR value, and N is the number of sampling points.

### 2.5. Feature Importance Analysis and Data Visualization

To interpret the contributions of individual environmental factors in the stacked model, SHAP v.0.48.0 was employed. For each base learner in the first layer, SHAP values were computed using TreeExplainer for tree-based models (LGBM, GBM, AdaBoost, and CatBoost) and KernelExplainer for non-tree models (SVR). In the second layer, the meta learner was explained by computing SHAP values for the meta-features, which are the predictions generated by the base learners, thereby quantifying each base learner’s contribution to the final prediction. Finally, global SHAP values were calculated to assess the overall influence of original input features on the stacked model’s output. The data were visualized using the matplotlib module (version 3.10.6) in Python (version 3.13.7).

## 3. Results and Discussion

### 3.1. Distinct Microbial Community Diversity and Structures in Three Anammox Systems

Microbial community composition across three distinct anammox systems was investigated using a total of 206 16S rRNA gene sequencing datasets obtained from 19 previous studies.

**Figure 2:**
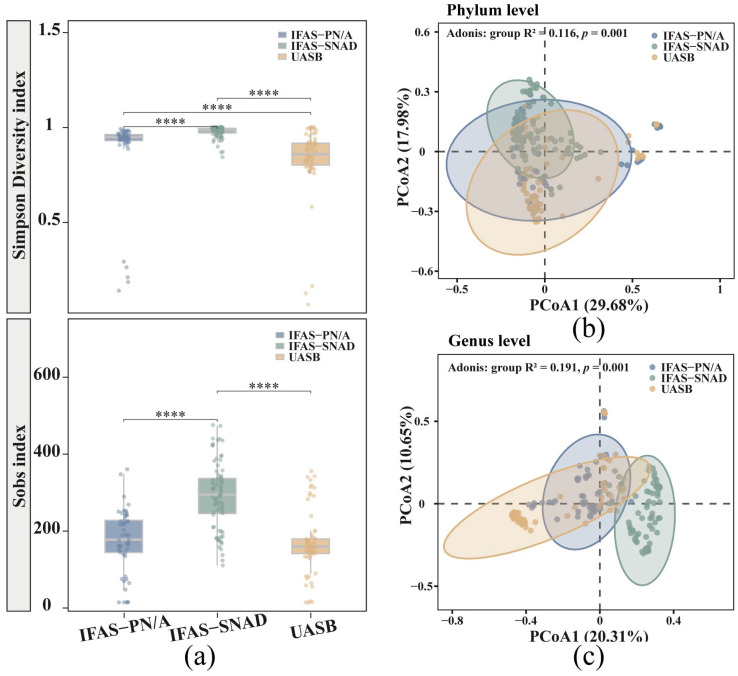
A comprehensive analysis of microbial communities in three different anammox systems. (**a**) Comparison of the Simpson Diversity and Sobs index among the three systems (**** *p* < 0.0001); (**b**) PCoA of microbial community within three systems based on phylum level Bray–Curtis distance; (**c**) PCoA of microbial community within three systems based on genus level Bray–Curtis distance.

As shown in Figure 2a, the Mann–Whitney U test revealed significant differences in the Simpson Diversity index in three systems (*p* < 0.0001). Among them, the IFAS-SNAD system exhibited the highest average Simpson Diversity index of 0.96, suggesting a greater diversity in this system’s microbial community. In addition, the IFAS-PN/A and UASB systems showed a lower average Simpson Diversity index of 0.86 and 0.82, respectively. In addition, the Sobs index provided a more intuitive representation of the differences in microbial richness across the three systems, and the IFAS-PN/A and UASB systems had lower microbial richness with significant differences compared to IFAS-SNAD (*p* < 0.0001). These findings illustrated that higher microbial diversity as well as richness were possessed by the IFAS-SNAD microbial community compared with IFAS-PN/A and UASB systems. In addition, principal coordinates analysis (PCoA) showed the difference in microbial community composition among three systems at the phylum and genus levels (Figure 2b,c). The Adonis test revealed that the differences in microbial community structure were particularly significant at two levels (*p* = 0.001), with more significant differences observed at the genus level.

### 3.2. Differential Abundance of Taxonomic Compositions

**Figure 3:**
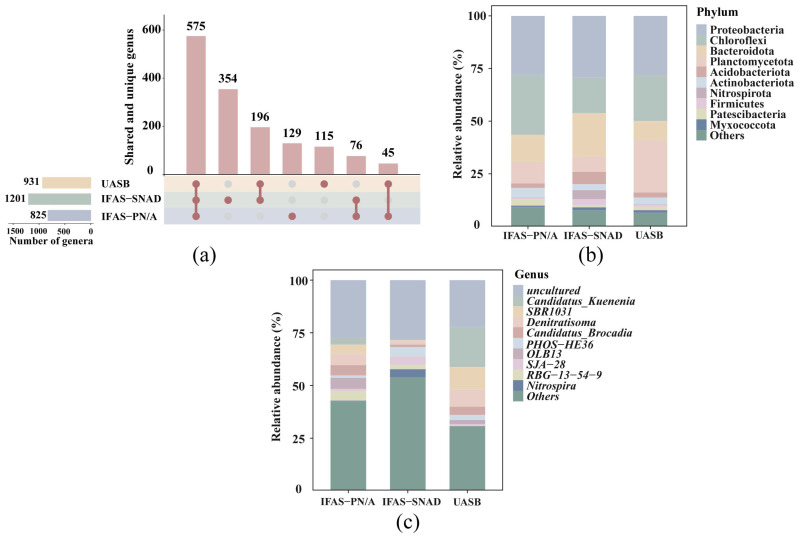
Comparison of bacteria genera across three distinct systems. (**a**) Venn diagram illustrating the shared and unique genus. Red dots represent the number of shared genera among systems (vertical positions correspond to values in the bar chart above). The number of dots connected by lines indicates the number of systems sharing the genera. (**b**) The top ten phylum with the highest relative abundance in three different systems; (**c**) The top ten genus with the highest relative abundance in three different systems.

A total of 825, 1201, and 931 genera were detected in IFAS-PN/A, IFAS-SNAD, and UASB systems, respectively (Figure 3a). IFAS-SNAD system exhibited the highest number of unique bacterial genera (354), while UASB system had the lowest (115). Additionally, 575 genera were found to be common across all three systems, suggesting their widespread presence within these systems. Only a limited number of genera overlapped between different systems, highlighting the distinctive microbial community structures characteristic of each treatment process.

Furthermore, the top ten phyla and the top ten genera with the highest relative abundance in three different systems were presented in Figure 3b,c. At the phylum level, microbial communities were primarily composed of Proteobacteria (28.1–29.4%), Chloroflexi (16.8–28.4%), Bacteroidota (9.3–20.3%)*,* and Planctomycetota (7.5–24.6%), which collectively accounted for more than 74.1% of total abundance. Among these, the Proteobacteria with denitrification and nitrification capabilities and the Bacteroidetes with the ability to degrade complex organic matter exhibited the highest relative abundances in the IFAS-SNAD system [42,43], at 29.4% and 20.3%, respectively. The high richness of these two phyla was consistent with the SNAD process integrating nitrification, anammox, and denitrification [44]. Specifically, the increased richness of these phyla was attributed to the presence of trace DO and more complex organic matter environments, enabling functions such as simultaneous denitrification within the IFAS-SNAD system [45]. Chloroflexi had been found to exhibit high relative abundance in the three systems. The higher abundance of Chloroflexi could be attributed to their role in scavenging macromolecules derived from dead anammox biomass and these filamentous bacteria, thereby maintaining and strengthening the structural integrity of anammox consortia [46,47,48]. Planctomycetota, which contains AnAOB, was found to account for 10.2%, 7.5%, and 24.6% in IFAS-PN/A, IFAS-SNAD, and UASB systems, respectively. Notably, Planctomycetota was significantly more abundant in the UASB system (24.6%) compared to the other two systems, reflecting that nitrogen removal of anammox process accounted for a larger proportion in the UASB system.

At the genus level, notable differences were observed in the composition and abundance of AnAOB in the three systems. In IFAS-PN/A system, *Candidatus_Brocadia* (5.0%) was the most abundant, while the UASB system was dominated by *Candidatus_Kuenenia* (19.0%). However, the relative abundance of *Candidatus_Brocadia* (1.2%) and *Candidatus_Kuenenia* (0.4%) in IFAS-SNAD system was lower compared to the other two systems. It was attributed to simultaneous occurrence of nitrification, anammox, and denitrification in the IFAS-SNAD system, which promoted the growth of bacteria such as nitrifying bacteria and denitrifying bacteria, leading to a reduced relative abundance of AnAOB. *SBR1031* and *Denitratisoma* were notably abundant in both UASB and IFAS-PN/A systems. Previous studies showed that *SBR1031* participated in organic matter degradation [49], while *Denitratisoma*, a well-defined denitrifier, cooperated with AnAOB for nitrogen removal in anammox systems [50], indicating their key roles in both systems. The unique enrichment of *PHOS-HE36* in the IFAS-SNAD system, accounting for approximately 4.53%, was noteworthy. It has been speculated that these obligate heterotrophic bacteria might be involved in the denitrification process [51]. And the relative abundance of *Nitrospira* (NOB) was also higher than in the other two systems. This observation could be explained by the evolutionary background of *Nitrospira*, which originated from microaerophilic or anaerobic ancestors, and these organisms possess the ability to utilize simple organic molecules, enabling a mixotrophic mode of growth when substrate availability is restricted [52]. Furthermore, the high abundance of uncultured genus across all systems revealed the potential diversity yet to be functionally characterized [53].

### 3.3. Microbial Network Analysis

#### 3.3.1. Network Topological Features

**Figure 4:**
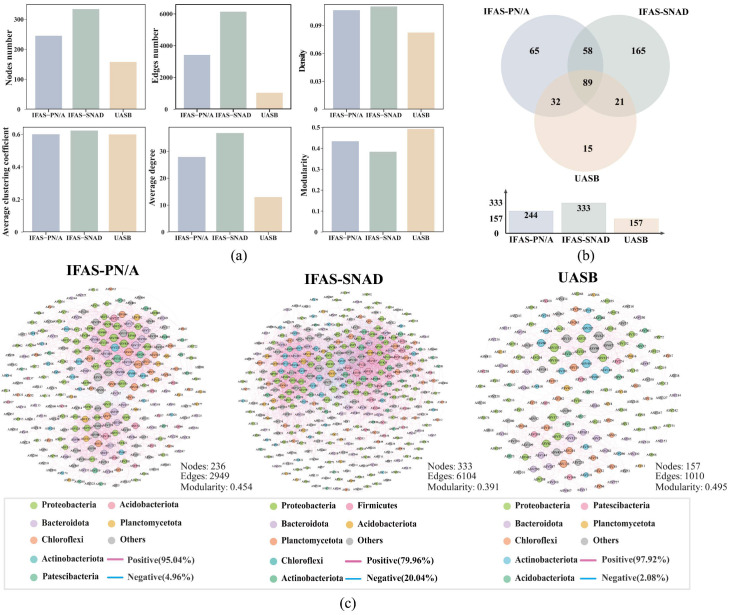
Microbial network analysis of ASVs across different systems. (**a**) The differences of network topological parameters across different systems; (**b**) Venn diagram of nodes in three networks; (**c**) Network analysis of ASVs in different systems. Each node represents an ASV, with node size proportional to its degree and color denoting phylum.

Network analysis was conducted to investigate the distinct interactions within three anammox systems (Figure 4). The network topological features of the three systems were provided in Figure 4a. The resulting networks contained 157–333 nodes and 1010–6104 edges with the average degree (avgK) from 12.866 to 36.661. The avgK, representing the average number of connections per node, was highest in the IFAS-SNAD network (36.661), while lowest in the UASB network (12.866). Both the average clustering coefficient (avgCC) and density, which measure network compactness, were lowest in the UASB system and highest in the IFAS-SNAD system. In addition, the modularity, which indicates the strength of division of a network into modules, was the highest in the UASB network (0.495), while the lowest was in the IFAS-SNAD network (0.391). These observations suggested that the IFAS-SNAD system possessed a more tightly connected and complex symbiotic network. In contrast, the UASB system showed a simpler and more modular network. These differences in features directly reflected the variations in microbial interaction among the three systems.

#### 3.3.2. Network Structure in the Three Systems

The constructed three microbial networks were visualized in Figure 4c, offering insights into the different network structure across three systems. The majority of network nodes were taxonomically classified within seven primary phyla, including Proteobacteria (26.64–30.03%), Bacteroidota (10.51–16.56%), Chloroflexi (8.71–12.74%), and Planctomycetota (4.46–8.71%), among others. The proportion of individual phyla represented in the networks varied notably from their relative abundances in the corresponding microbial communities. In particular, nodes belonging to Planctomycetota in the UASB networks only accounted for 4.46%, a marked contrast to their relative abundance (24.6%) in the microbial community. While the proportion of nodes related to Proteobacteria remained consistent at approximately 45.8% across three networks, suggesting the potential dominant function of Proteobacteria in maintaining the structural stability of anammox system networks.

Furthermore, 89 nodes were found to be shared among the three microbial networks (Figure 4b), with the majority belonging to Proteobacteria (23.8%), Bacteroidota (20.4%), and Chloroflexi (15.9%). And 65, 165, and 15 distinct nodes were observed in the IFAS-PN/A, IFAS-SNAD, and UASB networks, respectively. The networks of the two IFAS systems exhibited higher node diversity than the UASB system, consistent with the conclusions from the aforementioned microbial community analysis. These findings indicated the two IFAS microbial networks exhibited greater complexity and species diversity than the UASB network. The three networks comprised 10,504 edges. Of them, 93.4% of the edges (9809 edges) appeared only once across the three networks, which could be random or temporary correlations between species, displaying a significant difference in interactions among networks. Only 11 edges were present across three systems, and 9 of 11 edges reflected positive correlations. Moreover, the four related taxa (ASVs 130, 313, 353, and 358) involved in these nine positive connections were all derived from the same module, suggesting particularly strong interrelationships among them.

In addition, three anammox systems were all dominated by positive relationships, accounting for 95.04% (IFAS-PN/A), 79.96% (IFAS-SNAD), and 97.92% (UASB) of the total edges. It indicated that cooperative relationships were predominant in the three networks. And the resulting three networks generated 7, 8, and 6 modules with the modularity of 0.454, 0.391, and 0.495, respectively, illustrating that microorganisms tended to cluster together to maintain the stability of the network structure. It was also found that the M2 (IFAS-PN/A), M1 (IFAS-SNAD), and M1 (UASB) were the largest modules in these three networks, containing 102, 149, and 82 nodes, respectively (Figure S2). Among them, *Candidatus_Brocadia*, *SM1A02*, and *Candidatus_Kuenenia*, regarded as typical or potential AnAOB, were observed in the IFAS-PN/A, IFAS-SNAD, and UASB networks, respectively, suggesting that these modules performed the primary function of the anammox process and AnAOB required close interactions with other species during this process. Furthermore, the proportion of positive correlations between AnAOB and other taxa was lower in the IFAS-SNAD system (55.93%) compared to the IFAS-PN/A system (91.76%) and the UASB system (72.22%). The IFAS-SNAD network exhibited higher taxonomic competition, consistent with the fact that the organic environment favored the rapid proliferation of heterotrophic bacteria, which might compete with AnAOB for substrates and thereby inhibit their growth [54].

### 3.4. The Prediction of the Impact of Environmental Factors on NRR Using ML

In this study, datasets from three systems (IFAS-PN/A, IFAS-SNAD, and UASB) were used for model training and testing, and model performance was evaluated using statistical metrics. The stacked models combined with SHAP analysis were used to predict the nitrogen removal rate (NRR) and to interpret the influence of environmental factors on NRR.

#### 3.4.1. Evaluation of Model Performance

**Figure 5:**
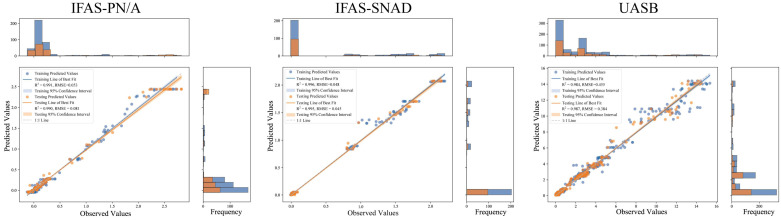
Predictive performance of ST-RF model for NRR in the three systems. In this bar chart, blue denotes training set data and orange denotes testing set data. These colors represent predicted result distributions for the model’s training and testing phases.

The evaluation metrics for the training and testing datasets of five base models and the stacked models ST-RF and ST-XGB were showed in Table 1. For the IFAS-PN/A testing dataset, the R^2^, MAE, and RMSE values of individual ML models ranged from 0.787 to 0.988, 0.060 to 0.217, and 0.090 to 0.378, respectively, while the average values of the corresponding metrics for stacked models were 0.990, 0.041, and 0.091, respectively. It was found that the performance of the stacked models was superior to that of the individual ML models, with the best model, ST-RF, showing R^2^, MAE, and RMSE values of 0.990, 0.042, and 0.081, respectively. Similar results were observed in the testing datasets in IFAS-SNAD and UASB systems. Furthermore, the model evaluation metrics for the training datasets’ performance comparison across the three systems aligned with the aforementioned testing dataset results. These findings collectively demonstrated that the predicted NRR values from the ST-RF model consistently agreed with the actual NRR values (Figure 5), achieving significantly higher prediction accuracy than other models. The stacked models ST-RF effectively enhanced prediction precision and reduced prediction errors.

#### 3.4.2. Identification of Key Environmental Factors

**Figure 6:**
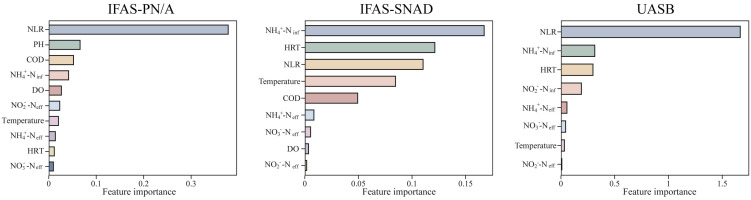
Final investigation on the importance of environmental factor features. The *x*-axis represented the importance of each feature, which meant the degree of contribution of each feature to the model prediction results.

Given the higher predictive accuracy of the ST-RF model, the SHAP method was applied to interpret the ST-RF model and obtain the importance ranking of each environmental factor in the three systems, with the results shown in Figure 6.

In IFAS-PN/A system, the five features with the greatest impact on NRR were NLR, PH, chemical oxygen demand (COD), NH_4_^+^-N_inf_, and DO. Specifically, pH and DO regulated the selection of AOB and NOB and thus determined the availability of nitrite [55], whereas NH_4_^+^-N_inf_ and NLR were directly related to substrate supply. At the same time, COD needed to be maintained within an optimal range that permitted limited heterotrophic activity to support denitrification while avoiding suppression of autotrophic anammox pathways [56]. In IFAS-SNAD system, the five features with the greatest impact on NRR were NH_4_^+^-N_inf_, hydraulic retention time (HRT), NLR, temperature, and COD. Among these, appropriate HRT and temperature provided the necessary conditions for the concurrent expression of these pathways. Meanwhile, NH_4_^+^-N_inf_ and NLR determined the nitrite supply required to sustain both nitrification and anammox activity. Compared with the two IFAS systems, NRR in UASB system was influenced not only by NLR and HRT but also by influent concentrations (NH_4_^+^-N_inf_ and NO_2_^−^-N_inf_). A previous study reported that the performance and stability of UASB-anammox systems were mainly controlled by substrate concentration and hydraulic conditions, with excessive nitrogen loading rates or nitrite levels beyond tolerance thresholds causing substrate inhibition and reduced nitrogen removal efficiency [57]. These results suggested that NRR of different systems depended on the control of key environmental factors, and the importance of influencing factors depended on the system type.

### 3.5. Impacts of Environmental Factors on Bacterial Communities

Based on the analysis of microbial community differences and the environmental factor importance ranking derived using SHAP in the IFAS-PN/A, IFAS-SNAD, and UASB systems, specific environmental factors were found to be associated with bacterial community composition changes in the three systems.

In the IFAS-PN/A system, SHAP analysis identified NLR, pH, COD, NH_4_^+^-N_inf_, and DO as the most influential environmental factors affecting NRR. Biologically, slightly low DO and optimal pH favor partial nitritation by suppressing NOB, thereby ensuring a steady nitrite supply to AnAOB. NH_4_^+^-N_inf_ and NLR directly determined substrate supply, and moderate NLR could promote the selective enrichment of AnAOB, whereas excessive NLR could cause the inhibition of their growth [58]. In addition, COD concentration also served as a pivotal regulatory factor. A previous study has found that heterotrophic denitrifiers could compete with AnAOB for nitrite under high COD conditions, thereby inhibiting the activity of AnAOB and reducing nitrogen removal efficiency. Even when AnAOB remain dominant, this competition might drive their metabolism toward organic substrate utilization rather than ammonium conversion, primarily impairing nitrogen metabolism [59]. The high relative abundance of *Candidatus_Brocadia* (5.0%) in this system was a direct consequence of regulation by these environmental factors. Previous studies demonstrated that *Candidatus_Brocadia* had the ability to thrive in partial nitrification environments [60,61], and network analysis results also revealed *Candidatus_Brocadia* occupied a central position in the largest network module and primarily exhibited cooperative relationships with other taxa, highlighting the important role in IFAS-PN/A system.

In the IFAS-SNAD system, the SHAP revealed that NRR was dependent on NH_4_^+^-N_inf_, HRT and NLR, while other features, such as COD and temperature, also exerted a certain degree of influence on the NRR. This finding reflected the multifaceted nature of the SNAD process, which required a balance between aerobic, anoxic, and anaerobic conditions to support simultaneous nitrification, denitrification, and anammox. A previous study had demonstrated that intermittent aeration and organic load control were crucial for maintaining the balance of nitrification, anammox, and denitrification processes within SNAD systems, while also highlighting the importance of biofilm carriers for retaining slow-growing AnAOB [62]. This balance was further reflected in the distinct microbial community structure. The relatively long HRT supported the retention and enrichment of AnAOB while provided sufficient contact time for the growth of heterotrophic denitrifiers. Especially, the enrichment of denitrifying bacteria *PHOS-HE36* and nitrifying *Nitrospira* (NOB) ensured a sufficient supply of nitrite for the anammox process. Network analysis further revealed that AnAOB appeared in the largest module, and exhibited competitive relationships with most taxa. This might be attributed to competition for substrates between heterotrophic bacteria and AnAOB, thereby inhibiting the growth of AnAOB. Thus, these environmental factors could directly influence the microbial community, which in turn mediated the nitrogen removal performance of the SNAD process through complex microbial interactions.

In the UASB system, the model indicated a strong dependence of NRR on NLR, with other environmental factors showing less impact. The overwhelming dominance of NLR as the primary environmental driver would impose extreme selective pressure that led to the enrichment of dominant microorganisms. It was consistent with the significantly higher relative abundance of *Candidatus_Kuenenia* compared to other species in this study. A similar finding has been obtained from an earlier investigation, for example, *Candidatus_Kuenenia* exhibited a high rate of protein synthesis and metabolic activity, making it particularly well-suited for high NLR and rapid growth rates [63]. According to the research conducted by Kim et al. [64], the granular UASB system as a nitrogen removal system showed excellent performance compared with the biofilm system at high NLR, further substantiating the efficient denitrification capabilities of UASB system. Our preceding analyses of species diversity and network structure also supported this. The UASB system exhibited low microbial diversity with a high abundance of AnAOB, and the corresponding microbial network was characterized by low modularity and fewer nodes, indicating a highly specialized anammox process within the system.

In summary, different environmental parameters significantly influenced the microbial community structure, which directly determined the functional characteristics of each reactor system. IFAS-SNAD system was shaped by coordinated regulation of multiple environmental factors. This system exhibited higher microbial richness and more complex, connected co-occurrence network, thereby enhancing functional stability and promoting synergistic nitrogen removal through the SNAD pathway. In contrast, the IFAS-PN/A system exhibited intermediate diversity and network complexity, which was consistent with its functional focus on the synergistic interaction between AOB and *Candidatus_Brocadia*, maintained by the factors such as DO and pH. Meanwhile, the low-diversity but highly modular network in the UASB system reflected a specialized community dominated by *Candidatus_Kuenenia*, which could enhance the NRR under high NLR but might be more vulnerable to environmental factors.

## 4. Conclusions

This study systematically compared the microbial community structure, microbial networks, and nitrogen removal performance across three anammox systems (UASB, IFAS-PN/A, and IFAS-SNAD). The results revealed different microbial diversity and composition among the systems, with IFAS-SNAD exhibiting the highest diversity and functional complexity, while IFAS-PN/A and UASB displayed lower diversity and were dominated by *Candidatus_Brocadia* and *Candidatus_Kuenenia*, respectively. Network analysis further indicated that IFAS-SNAD possessed a more complex microbial interaction network, whereas UASB showed a simpler network. Furthermore, ML analysis showed that stacked models consistently outperformed single models in predicting NRR accuracy, with the ST-RF model demonstrating the highest predictive precision (R^2^ > 0.987). And SHAP analysis demonstrated that the impact of different environmental factors on NRR depended on the type of system. Notably, the operational differences across the three anammox systems reflect practical application diversity, and these differences were fully accounted for by identifing system-specific environmental factors using ML and SHAP analysis.

Overall, the bioinformatics and ML methods are conducted in this study to examine variations in microbial communities among the three systems and to assess how environmental parameters influence NRR. The integration of high-throughput sequencing with ML presents a powerful and data-driven pathway for enhancing water pollution control, providing theoretical support for constructing more efficient ecological engineering systems for wastewater treatment. However, the model in this study has only been internally validated, and external validation is recommended to further confirm its generalizability and practical applicability.

## Figures and Tables

**Table 1:** Comparison of evaluation metrics across models.

		Training Dataset			Testing Dataset		
	Models	R^2^	MAE	RMSE	R^2^	MAE	RMSE
**IFAS-PN/A**	AdaBoost	0.986	0.046	0.065	0.988	0.060	0.090
	CatBoost	0.876	0.118	0.194	0.843	0.186	0.325
	LGBM	0.895	0.088	0.179	0.848	0.160	0.319
	GBM	0.794	0.150	0.250	0.787	0.217	0.378
	SVR	0.921	0.098	0.155	0.901	0.155	0.258
	**ST-RF**	0.991	0.028	0.053	**0.9** **90**	**0.0** **42**	**0.0** **81**
	ST-XGB	0.991	0.027	0.050	0.989	0.040	0.100
**IFAS-SNAD**	AdaBoost	0.994	0.028	0.058	0.993	0.026	0.056
	CatBoost	0.903	0.173	0.230	0.903	0.159	0.206
	LGBM	0.937	0.153	0.186	0.935	0.140	0.169
	GBM	0.796	0.285	0.334	0.788	0.262	0.305
	SVR	0.974	0.093	0.119	0.969	0.092	0.116
	**ST-RF**	0.996	0.021	0.048	**0.99** **5**	**0.0** **22**	**0.0** **45**
	ST-XGB	0.993	0.026	0.061	0.994	0.024	0.053
**UASB**	AdaBoost	0.980	0.298	0.509	0.977	0.280	0.516
	CatBoost	0.883	0.816	1.220	0.899	0.753	1.069
	LGBM	0.929	0.609	0.950	0.936	0.571	0.851
	GBM	0.781	1.140	1.669	0.795	1.061	1.527
	SVR	0.811	0.669	1.551	0.840	0.590	1.347
	**ST-RF**	0.984	0.222	0.455	**0.98** **7**	**0.19** **8**	**0.** **384**
	ST-XGB	0.979	0.239	0.520	0.981	0.222	0.468

## Data Availability

The original contributions presented in this study are included in the article/Supplementary Material. Further inquiries can be directed to the corresponding author.

## Supplementary Materials

The following supporting information can be downloaded at: https://www.mdpi.com/article/10.3390/microorganisms13122795/s1, Figure S1. Literature Search Diagram.; Figure S2. Microbial network analysis of ASVs across different systems organized by different modules. Each node represents an ASV, with node size proportional to its degree and color denoting different modules.; Table S1. Information of three types of systems analyzed in this study; Supplementary Material S2. Environmental factor data collected for three systems in this study. Refs. [65,66,67,68,69,70,71,72,73,74,75,76,77,78,79,80] can be found in Supplementary Materials.

## Abbreviations

The following abbreviations are used in this manuscript:

Anammox	Anaerobic ammonia oxidation
NH_4_^+^-N	Ammonium nitrogen
NO_2_^−^-N	Nitrite nitrogen
N_2_	Nitrogen gas
IFAS	Integrated fixed-film activated sludge
PN/A	Partial nitritation/anammox
SNAD	Simultaneous nitrification, anammox, and denitrification
UASB	Upflow anaerobic sludge blanket
IFAS-PN/A	Integrated fixed-film activated sludge-partial nitritation/anammox
IFAS-SNAD	Integrated fixed-film activated sludge-simultaneous nitrification, anammox, and denitrification
ML	Machine learning
SHAP	SHapley Additive exPlanations
PCoA	Principal Coordinates Analysis
NCBI	National Center for Biotechnology Information
ASV	Amplicon sequence variant
AnAOB	Anammox bacteria
AOB	Ammonium-oxidizing bacteria
NOB	Nitrite-oxidizing bacteria
DO	Dissolved oxygen
TN	Total nitrogen
HRT	Hydraulic retention time
COD	Chemical oxygen demand
ANN	Artificial Neural Network
SVR	Support Vector Regression
LGBM	Light Gradient Boosting Machine
RF	Random Forest
XGBoost	Extreme Gradient Boosting
CatBoost	Categorical Boosting
AdaBoost	Adaptive Boosting
GBM	Gradient Boosting Machine
ST-XG	Stacked-XGBoost
ST-RF	Stacked-RF
NRR	Nitrogen Removal Rate
NLR	Nitrogen Loading Rate
NRE	Nitrogen removal efficiency
R^2^	Determination coefficients
RMSE	Root mean square error
MAE	Mean absolute error
avgK	Average degree
avgCC	Average clustering coefficient
