# Supplementary material for: A Comprehensive Analysis of Microbial Community and Nitrogen Removal Rate Predictions in Three Anammox Systems
Source: Microorganisms. 2025 Dec 8;13(12):2795. doi: 10.3390/microorganisms13122795 (PMC12735504; doi:10.3390/microorganisms13122795)
Supplement: Supplementary file 1 [file microorganisms-13-02795-s001.zip › Supplementary Material S1.pdf]

Supplementary Material

Table S1. Information of three types of systems analyzed in this study

Type	Sequencing Region	Sample Number	Accession numbers	Reference
IFAS-PN/A	V3- V4	9	SRR6891810- SRR6891818	[1]
	V3- V4	16	SRR9943601- SRR9943606<br>SRR9943609- SRR9943618	[2,3]
	V3- V4	19	SRR24031802-SRR24031811<br>SRR24031813, SRR24031824<br>SRR24031817- SRR24031819<br>SRR24031826- SRR24031827<br>SRR24031835- SRR24031836	[4]
	V3- V4	2	SRR4297636- SRR4297637	[5]
	V3- V4	6	SRR14923867<br>SRR16532604- SRR16532608	[6]
	V3- V4	3	SRR10811332- SRR10811334	[7]
IFAS-SNAD	V3- V4	6	SRR6037377- SRR6037382	[8]
	V3- V4	7	SRR10199657<br>SRR10230798- SRR10230803	[9]
	V4- V5	73	SRR10850409-SRR10850411<br>SRR10850413-SRR10850414<br>SRR10850417-SRR10850433<br>SRR10850435-SRR10850437<br>SRR10850439-SRR10850440<br>SRR10850442, SRR10850462, SRR10850464<br>SRR10850444-SRR10850459<br>SRR10850466-SRR10850492	[10]
UASB	V3- V4	1	SRR2960312	[11]
	V3- V4	3	SRR13492910- SRR13492912	[12]
	V3- V4	35	SRR19392144- SRR19392178	[13]
	V3- V4	6	SRR30225396- SRR30225401	[14]
	V3- V4	4	SRR21284478- SRR21284481	[15]
	V4- V5	2	SRR2553316, SRR2510538	[16]
	V4	1	SRR4381493	[17]
	V4	7	SRR18677381- SRR18677383<br>SRR29278021- SRR29278022<br>SRR29278026- SRR29278027	[18]
	V3- V4	6	SRR5135096, SRR5135128<br>SRR5135130- SRR5135133	[19]

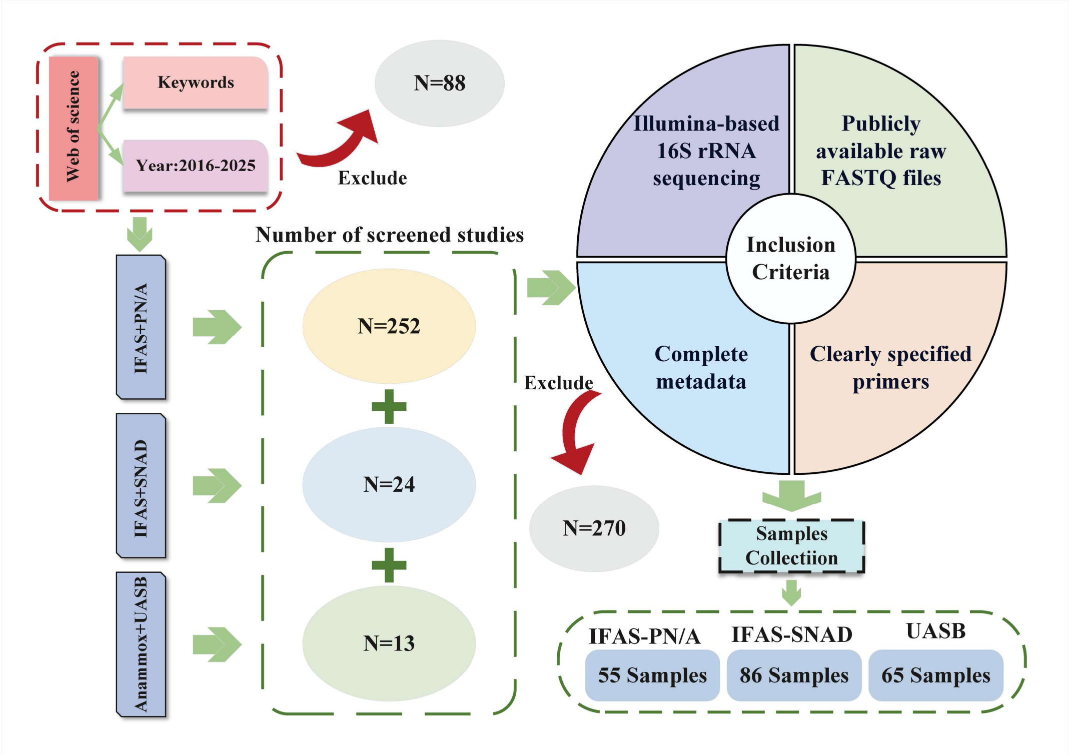

Figure S1. Literature Search Diagram

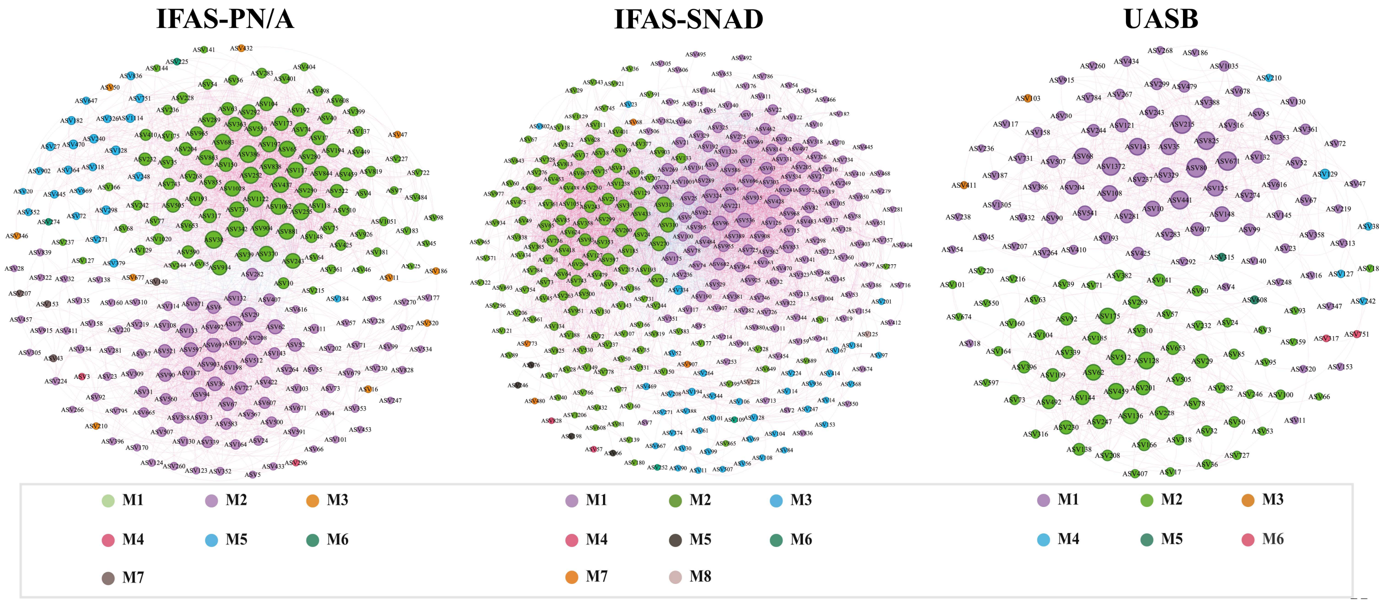

Figure S2. Microbial network analysis of ASVs across different systems organized by different modules. Each node represents an ASV, with node size proportional to its degree and color denoting different modules.

References

1. Yang, S.; Peng, Y.; Zhang, L.; Zhang, Q.; Li, J.; Wang, X. Autotrophic Nitrogen Removal in an Integrated Fixed-Biofilm Activated Sludge (IFAS) Reactor: Anammox Bacteria Enriched in the Flocs Have Been Overlooked. *Bioresource Technology* **2019**, *288*, 121512, doi:10.1016/j.biortech.2019.121512.
2. Yang, S.; Peng, Y.; Zhang, S.; Han, X.; Li, J.; Zhang, L. Carrier Type Induces Anammox Biofilm Structure and the Nitrogen Removal Pathway: Demonstration in a Full-Scale Partial Nitritation/Anammox Process. *Bioresource Technology* **2021**, *334*, 125249, doi:10.1016/j.biortech.2021.125249.
3. Han, X.; Zhang, S.; Yang, S.; Zhang, L.; Peng, Y. Full-Scale Partial Nitritation/Anammox (PN/A) Process for Treating Sludge Dewatering Liquor from Anaerobic Digestion after Thermal Hydrolysis. *Bioresource Technology* **2020**, *297*, 122380, doi:10.1016/j.biortech.2019.122380.
4. Yang, M.; Li, J.; Li, Z.; Peng, Y.; Zhang, L. Enhancing Anammox Bacteria Enrichment in Integrated Fixed-Film Activated Sludge Partial Nitritation/Anammox Process via Floc Retention Control. *Bioresource Technology* **2024**, *391*, 129938, doi:10.1016/j.biortech.2023.129938.
5. Yang, Y.; Zhang, L.; Cheng, J.; Zhang, S.; Li, B.; Peng, Y. Achieve Efficient Nitrogen Removal from Real Sewage in a Plug-Flow Integrated Fixed-Film Activated Sludge (IFAS) Reactor via Partial Nitritation/Anammox Pathway. *Bioresource Technology* **2017**, *239*, 294–301, doi:10.1016/j.biortech.2017.05.041.
6. Ren, S.; Wang, Z.; Jiang, H.; Li, X.; Zhang, Q.; Peng, Y. Efficient Nitrogen Removal from Mature Landfill Leachate in a Step Feed Continuous Plug-Flow System Based on One-Stage Anammox Process. *Bioresource Technology* **2022**, *347*, 126676, doi:10.1016/j.biortech.2022.126676.
7. Liu, W.; Shen, C.; Liu, C.; Zhang, S.; Hao, S.; Peng, Y.; Li, J. Achieving Stable Mainstream Nitrogen and Phosphorus Removal Assisted by Hydroxylamine Addition in a Continuous Partial Nitritation/Anammox Process from Real Sewage. *Science of The Total Environment* **2021**, *794*, 148478, doi:10.1016/j.scitotenv.2021.148478.
8. Wang, C.; Liu, S.; Xu, X.; Zhang, C.; Wang, D.; Yang, F. Achieving Mainstream Nitrogen Removal through Simultaneous Partial Nitrification, Anammox and Denitrification Process in an Integrated Fixed Film Activated Sludge Reactor. *Chemosphere* **2018**, *203*, 457–466, doi:10.1016/j.chemosphere.2018.04.016.
9. Du, Y.; Yu, D.; Wang, X.; Zhen, J.; Bi, C.; Gong, X.; Zhao, J. Achieving Simultaneous Nitritation, Anammox and Denitrification (SNAD) in an Integrated Fixed-Biofilm Activated Sludge (IFAS) Reactor: Quickly Culturing Self-Generated Anammox Bacteria. *Science of The Total Environment* **2021**, *768*, 144446, doi:10.1016/j.scitotenv.2020.144446.
10. Roots, P.; Rosenthal, A.F.; Yuan, Q.; Wang, Y.; Yang, F.; Kozak, J.A.; Zhang, H.; Wells, G.F. Optimization of the Carbon to Nitrogen Ratio for Mainstream Deammonification and the Resulting Shift in Nitrification from Biofilm to Suspension. *Environ. Sci.: Water Res. Technol.* **2020**, *6*, 3415–3427, doi:10.1039/D0EW00652A.
11. Wang, Y.; Hu, X.; Jiang, B.; Song, Z.; Ma, Y. Symbiotic Relationship Analysis of Predominant Bacteria in a Lab-Scale Anammox UASB Bioreactor. *Environ Sci Pollut Res* **2016**, *23*, 7615–7626, doi:10.1007/s11356-015-6016-z.

12. Peng, Z.; Lei, Y.; Liu, Y.; Wan, X.; Yang, B.; Pan, X. Fast Start-up and Reactivation of Anammox Process Using Polyurethane Sponge. *Biochemical Engineering Journal* **2022**, *177*, 108249, doi:10.1016/j.bej.2021.108249.
13. Ya, T.; Huang, Y.; Wang, K.; Wang, J.; Liu, J.; Hai, R.; Zhang, T.; Wang, X. Functional Stability Correlates with Dynamic Microbial Networks in Anammox Process. *Bioresource Technology* **2023**, *370*, 128557, doi:10.1016/j.biortech.2022.128557.
14. Peng, Z.; Zhang, L.; Zhang, Q.; Li, X.; Peng, Y. Establishing a Two-Stage System to Efficiently Treat Real Domestic Sewage by Partial Nitrification-SBR and Air-Lift Anammox-UASB: Reactivating and Enhancing Anammox Bacteria to Optimize the Nitrogen Removal Performance. *Chemical Engineering Journal* **2025**, *506*, 160333, doi:10.1016/j.cej.2025.160333.
15. Ma, Y.; Wang, B.; Li, X.; Wang, S.; Wang, W.; Peng, Y. Enrichment of Anammox Biomass during Mainstream Wastewater Treatment Driven by Achievement of Partial Denitrification through the Addition of Bio-Carriers. *Journal of Environmental Sciences* **2024**, *137*, 181–194, doi:10.1016/j.jes.2023.03.002.
16. Cao, S.; Du, R.; Li, B.; Ren, N.; Peng, Y. High-Throughput Profiling of Microbial Community Structures in an ANAMMOX-UASB Reactor Treating High-Strength Wastewater. *Appl Microbiol Biotechnol* **2016**, *100*, 6457–6467, doi:10.1007/s00253-016-7427-6.
17. Song, Y.-X.; Liao, Q.; Yu, C.; Xiao, R.; Tang, C.-J.; Chai, L.-Y.; Duan, C.-S. Physicochemical and Microbial Properties of Settled and Floating Anammox Granules in Upflow Reactor. *Biochemical Engineering Journal* **2017**, *123*, 75–85, doi:10.1016/j.bej.2017.04.002.
18. Gao, M.; Dang, H.; Zou, X.; Yu, N.; Guo, H.; Yao, Y.; Liu, Y. Deciphering the Role of Granular Activated Carbon (GAC) in Anammox: Effects on Microbial Succession and Communication. *Water Research* **2023**, *233*, 119753, doi:10.1016/j.watres.2023.119753.
19. Jia, F.; Peng, Y.; Li, J.; Li, X.; Yao, H. Metagenomic Prediction Analysis of Microbial Aggregation in Anammox-dominated Community. *Water Environment Research* **2021**, *93*, 2549–2558, doi:10.1002/wer.1529.
